# Shotgun metagenome data of a defined mock community using Oxford Nanopore, PacBio and Illumina technologies

**DOI:** 10.1038/s41597-019-0287-z

**Published:** 2019-11-26

**Authors:** Volkan Sevim, Juna Lee, Robert Egan, Alicia Clum, Hope Hundley, Janey Lee, R. Craig Everroad, Angela M. Detweiler, Brad M. Bebout, Jennifer Pett-Ridge, Markus Göker, Alison E. Murray, Stephen R. Lindemann, Hans-Peter Klenk, Ronan O’Malley, Matthew Zane, Jan-Fang Cheng, Alex Copeland, Christopher Daum, Esther Singer, Tanja Woyke

**Affiliations:** 1 DOE Joint Genome Institute, 2800 Mitchell Drive, Walnut Creek, CA 94598 USA; 2 NASA Ames Research Center, Exobiology Branch, Moffett Field, CA 94035 USA; 3 Bay Area Environmental Research Institute, Moffett Field, CA 94035 USA; 4 Lawrence Livermore National Laboratory, Nuclear and Chemical Science Division, 7000 East Ave, Livermore, CA 94550-9234 USA; 5 Leibniz-Institut DSMZ-Deutsche Sammlung von Mikroorganismen und Zellkulturen GmbH, Inhoffenstraße 7B, 38124 Braunschweig, Germany; 6 Desert Research Institute, Division of Earth and Ecosystem Sciences, 2215 Raggio Pkwy, Reno, NV 89512 USA; 7 Purdue University, 610 Purdue Mall, West Lafayette, IN 47907 USA; 8 Newcastle University, School of Natural and Environmental Sciences, Ridley Building 2, Newcastle upon Tyne, NE1 7RU UK; 9 Lawrence Berkeley National Laboratory, 1 Cyclotron Road, Berkeley, CA 94720 USA

**Keywords:** DNA sequencing, Metagenomics, Data acquisition, Hardware and infrastructure

## Abstract

Metagenomic sequence data from defined mock communities is crucial for the assessment of sequencing platform performance and downstream analyses, including assembly, binning and taxonomic assignment. We report a comparison of shotgun metagenome sequencing and assembly metrics of a defined microbial mock community using the Oxford Nanopore Technologies (ONT) MinION, PacBio and Illumina sequencing platforms. Our synthetic microbial community BMock12 consists of 12 bacterial strains with genome sizes spanning 3.2–7.2 Mbp, 40–73% GC content, and 1.5–7.3% repeats. Size selection of both PacBio and ONT sequencing libraries prior to sequencing was essential to yield comparable relative abundances of organisms among all sequencing technologies. While the Illumina-based metagenome assembly yielded good coverage with few misassemblies, contiguity was greatly improved by both, Illumina + ONT and Illumina + PacBio hybrid assemblies but increased misassemblies, most notably in genomes with high sequence similarity to each other. Our resulting datasets allow evaluation and benchmarking of bioinformatics software on Illumina, PacBio and ONT platforms in parallel.

## Abstract (summary)

**Table:** 

Measurement(s)	metagenomic data • sequence_assembly
Technology Type(s)	ONT MinION • Illumina sequencing • PacBio RS II
Factor Type(s)	sequencing platform
Sample Characteristic - Organism	Bacteria
Sample Characteristic - Environment	mock community

Machine-accessible metadata file describing the reported data: 10.6084/m9.figshare.10260740

## Background & Summary

Accurate microbial community representation based on cultivation-independent genome sequencing methods has been one of the major challenges in microbial ecology and genomics since the onset of shotgun metagenome sequencing. Existing sequencing technologies display platform-specific biases depending on run mode and chemistry. These biases affect read length, data throughput, GC coverage bias, error rates, and the ability to resolve repetitive genomic elements^1–3^. The Oxford Nanopore Technology (ONT) MinION is the first commercially available sequencer that uses nanopores. In the MinION, nanopore sequencing discriminates individual nucleotides by measuring the change in electrical conductivity as DNA molecules pass through a biological pore^4^. The ONT MinION is a portable sequencing device generating maximum read lengths in excess of 100 kb with the potential to span long repeats, and at comparably low cost and high-speed (our test runs yielded 10–50 Gb in 48 hours). To date most published studies using the MinION technology focus on (i) whole genome sequencing (WGS) of organisms with existing reference genomes and on (ii) validating or resolving difficult regions or screens of target genes/gene regions in viral^5–12^, bacterial^5,6,13–28^, and eukaryotic^29–44^ genomes. Laver *et al*. compared ONT performance for three bacterial strains with % GC of ~29–71% and showed that the strain with highest % GC was underrepresented in the sequencing reads^45^. Various genome assemblies were shown to improve in hybrid approaches with Illumina reads^30^ and reached 99.5% nucleotide identity for a *de novo* assembly of *E*. *coli*^13^. To our knowledge, only two ONT shotgun metagenome studies exist, one of an environmental sample in which DNA was fragmented to ~510–840 bp and the resulting 2D reads (0–1200 bp) were mapped against a database of 400 bp gene fragments^46^, and the other of various low complexity mock communities comparing different long read classification tools^47^. To date, there has not been an ONT shotgun metagenome study that evaluates its long reads in the context of mapping accuracy, assembly contiguity, and overall community representation.

**Fig. 1:**
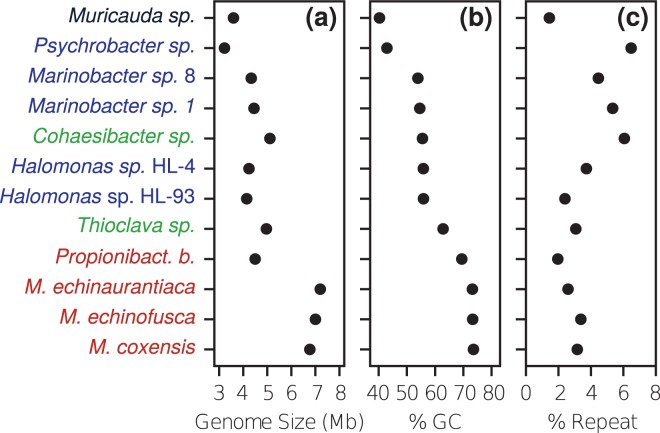
Microbial mock community strains display a large spread with respect to genome size, % GC and repeat content. Order was determined by GC content. Colors indicate phylum/class of each organism. Black = *Bacteroidetes*, Green = *Alphaproteobacteria*, Blue = *Gammaproteobacteria*, Red = *Actinobacteria*.

**Fig. 2:**
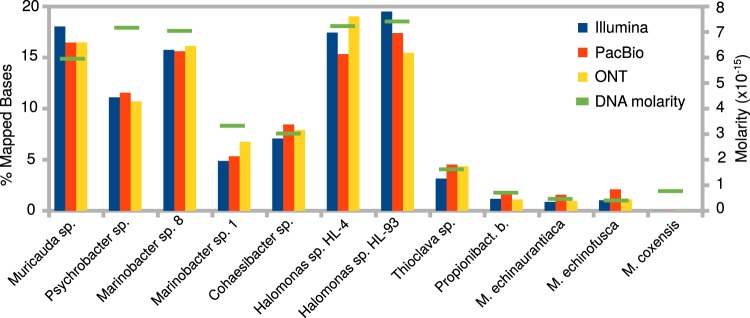
Distribution of mapped bases for each organism and technology, and molarity of each genome in the mock community. Molarities strongly correlate with mapped bases (Pearson correlation coefficient: 0.95) for all sequencing platforms. The total number of bases that mapped to *M*. *coxensis* was negligibly small.

We used a defined community (composed of a pool of separately extracted DNAs), BMock12, that includes 12 bacterial strains belonging to two phyla (*Actinobacteria* and *Flavobacteria*) and 2 proteobacterial classes (*Alpha-* and *Gammaproteobacteria*). Genomes from these taxa represent a breadth of genome sizes and range from low to high % GC with variable repeat fractions. Bmock12 includes three actinobacterial genomes of the genus *Micromonospora* characterized by high %GC content and high average nucleotide identity (ANI), which present challenges for assembly (Fig. 1, Table 1). Shotgun sequencing performance on ONT MinION was compared to other state-of-the-art platforms, Pacific Biosciences RS-II and Illumina HiSeq. 2500 (Table 2). Interestingly, we noticed a major impact of input DNA size selection during library preparation on the length distribution of mapped reads in ONT data, favoring the sequencing of shorter reads, which also resulted in a slightly skewed community structure (Figs. S1, S2). After size selection and removal of reads <10 kb, relative abundances of each organism were found to be comparable across all sequencing technologies, and equally correlated to molarity (Fig. 2, Tables S1, S2 and S3). Average % identity of both ONT and PacBio mapped reads was 85.9% (Figs. S3, S4). A negligible number of reads were mapped to *M*. *coxensis*, likely due to low input DNA concentration or quality, or as a result of pipetting error and/or inaccuracies in DNA quantification as was observed previously^48^. Therefore, this organism was omitted from the remainder of the analysis. Other disagreements between the distributions of % mapped bases and DNA molarity are likely due to these same noise factors.

**Table 1:** All genomes are available as improved high-quality drafts in the IMG database. See Fig. S1 for detailed statistics.

IMG Taxon ID	Organism	Phylum	Class
2615840527	*Muricauda* sp. ES.050	*Bacteroidetes*	*Flavobacteria*
2615840533	*Thioclava* sp. ES.032	*Proteobacteria*	*Alphaproteobacteria*
2615840601	*Cohaesibacter* sp. ES.047	*Proteobacteria*	*Alphaproteobacteria*
2615840646	*Propionibacteriaceae* bacterium ES.041	*Actinobacteria*	*Actinobacteria*
2615840697	*Marinobacter* sp. LV10R510-8	*Proteobacteria*	*Gammaproteobacteria*
2616644829	*Marinobacter* sp. LV10MA510-1	*Proteobacteria*	*Gammaproteobacteria*
2617270709	*Psychrobacter* sp. LV10R520-6	*Proteobacteria*	*Gammaproteobacteria*
2623620557	*Micromonospora echinaurantiaca* DSM 43904	*Actinobacteria*	*Actinobacteria*
2623620567	*Micromonospora echinofusca* DSM 43913	*Actinobacteria*	*Actinobacteria*
2623620609	*Micromonospora coxensis* DSM 45161	*Actinobacteria*	*Actinobacteria*
2623620617	*Halomonas* sp. HL-4	*Proteobacteria*	*Gammaproteobacteria*
2623620618	*Halomonas* sp. HL-93	*Proteobacteria*	*Gammaproteobacteria*

**Table 2:** Run information and statistics for each sequencing platform. Average quality score for Illumina reads was 35.3. Percent identity was calculated as E/(E + I + D + S), where, E, I, D, S represent exact matches, insertions, deletions and substitutions respectively.

	Illumina	PacBio	ONT
Instrument model	HiSeq-2500 1TB	RS-II	MinION
Sequencing chemistry	TruSeq SBS v.4	RSII v. C4	R9.4.1 (flow cell)
Run mode	2 × 150 indexed run	1 × 240 sequencing movie run	
Raw reads	426,735,646	389,806	187,507
Filtered reads	422,896,888	389,806	187,507
Filtered bases	63,384,840,109	2,583,337,248	3,737,495,058
Average insert/read size [bp]	302.70	6,627.00	19,932.60
Longest insert/read [bp]	625	45,165	145,720
Uniquely mapped reads	411,863,512	376,583	187,448
%Identity	99.8	85.9	85.9

**Fig. 3:**
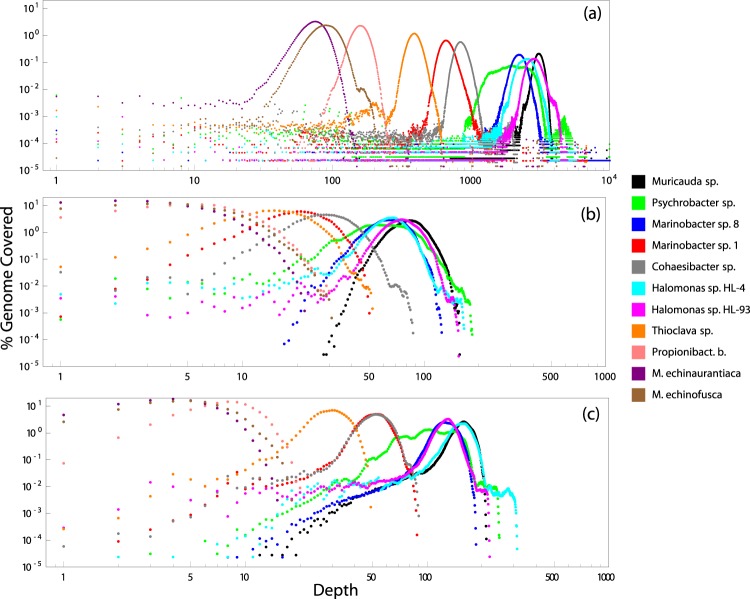
Genome coverage for all organisms and sequencing platforms displayed on a log-log scale. *M*. *coxensis* is excluded due to lack of mapped reads.

Although reads <10 kb were removed from ONT and PacBio datasets, the distribution of read lengths peaked at ~12 kb in ONT *vs*. ~5 kb in PacBio data, because PacBio sequences generally tend to favor shorter DNA molecules^49^ and likely because size selection for ONT was more successful (Fig. S5). The length distribution of reads mapped to each organism was found to be nearly the same within each sequencing platform (Fig. S6). PacBio and ONT reads displayed comparable distribution patterns of % genome coverage over sequencing depth (Figs. 3 and S7), and in contrast to Illumina reads, they did not show any notable GC bias (Fig. S8). Illumina sequences have previously been described to discriminate against GC-poor and GC-rich genomes and DNA regions^50–52^. Read mapping errors were mostly substitutions and deletions and, to a lesser degree, insertions for ONT, whereas PacBio errors were dominated by insertions (Figs. S9, S10).

Metagenome assembly was performed using (1) only Illumina reads, (2) Illumina and PacBio reads, or (3) Illumina and ONT reads. Illumina-only assemblies performed well and yielded at least 92.6% reference coverage (Table 3). 6 out of 11 Illumina-only genome assemblies displayed fewer misassemblies than the hybrid assemblies, which is likely due to the increased error rate in long reads. Misassemblies in hybrid assemblies were particularly high for the two *Halomonas* spp., which shared 99% ANI, indicating that hybrid assemblies might generally be challenged by the presence of strains of the same species, or more generally with high % ANI to each other. In the case of the two *Marinobacter* spp., which shared 85% ANI, only one of the two genomes generated few misassemblies in the hybrid assemblies (Tables 3 and S4). For all genomes, except that of *Proprionibacter bacterium*, contiguity improved greatly in the hybrid assemblies. In some hybrid assemblies, the total number of contigs was reduced by an order of magnitude. Illumina + ONT assemblies were less fragmented than Illumina + PacBio assemblies due to the longer average read lengths of the ONT reads (Fig. S11). ANI between genome pairs was the main factor determining the assembly quality (Table S4). Genomes that are closely related to others (particularly two *Halomonas* strains with 99% ANI) yielded lower quality assemblies (Table S5). This effect of strain heterogeneity on metagenome assembly has been previously reported through extensive benchmarking^53^. Similarly, genomes with high repeat content (*Psychrobacter*, *Cohaesibacter*, and both *Marinobacter* species) resulted in more fragmented assemblies as compared to others. Reference coverage was the same or better in hybrid assemblies with the exception of *Halomonas* sp. HL-4 (Table 3). Total aligned length was comparable between all sequencing technologies (Table S4). Genomes pairs with relatively high ANI (two *Halomonas* strains, *Marinobacter* sp. LV10R510-8, *Marinobacter* sp. LV10MA510-1, *M*. *echinaurantiaca* and *M*. *echinofusca*) displayed assembly lengths larger than their references, which resulted from contigs that mapped to more than one reference genome.

**Table 3:** Assembly statistics. NGA50 is the length of the shortest in the set of blocks of that length or longer covers at least 50% of the reference genome after alignment. Blocks are parts of contigs split at misassembly events.

Assemblies	Total Length [bp]			Reference Coverage [%]			No. Contigs		
	Illumina Only	Illumina + ONT	Illumina + PacBio	Illumina Only	Illumina + ONT	Illumina + PacBio	Illumina Only	Illumina + ONT	Illumina + PacBio
*Muricauda* sp.	3,579,780	3,596,256	3,590,644	99.6	99.8	99.9	14	3	2
*Thioclava* sp.	4,898,095	4,940,417	4,933,303	98.6	99.5	99.3	65	3	8
*Cohaesibacter* sp.	4,943,283	5,151,317	4,995,520	96.7	98.5	97.4	139	23	72
*Propionibact*. *b*.	4,495,270	4,495,756	4,495,756	100.0	100.0	100.0	2	2	2
*Marinobacter*. sp. 8	4,337,062	9,170,029	5,788,008	98.6	100.0	99.8	98	11	25
*Marinobacter* sp. 1	4,371,813	7,274,187	5,460,448	96.3	99.6	98.5	114	20	38
*Psychrobacter* sp.	3,173,207	3,229,220	3,224,906	97.4	99.2	99.1	122	41	44
*M*. *echinaurantiaca*	7,164,504	7,193,150	7,172,232	99.3	99.7	99.5	49	6	17
*M*. *echinofusca*	6,965,883	11,125,773	7,412,507	99.4	100.0	99.6	60	5	19
*Halomonas* sp. HL-4	4,007,588	7,577,667	4,772,878	92.6	99.3	85.0	477	56	149
*Halomonas* sp. HL-93	4,186,714	7,535,492	5,037,941	98.1	99.3	96.4	503	43	118
not_aligned	N/A	N/A	N/A	N/A	N/A	N/A	240	239	240

While arriving at the true community composition of complex microbiomes will remain challenging, current advancements in sequencing protocols have resulted in reduced bias, improved resolution, and more predictable error. Metagenomic sequence data from defined samples, such as MBARC-26^54^, HMP^55^, and the BMock12 data described here are critical to not only assess new or modified wet lab protocols^56^ and performance of sequencing platforms^57^, but also downstream analytical tools and pipelines used to derive biological insights from metagenome datasets^53,58^. While ONT had been primarily used for WGS for organisms with existing reference genomes, and hybrid assemblies as well as diagnostics, our study shows that shotgun metagenome data generated on the MinION yields community representation and improved genome assembly contiguity that is comparable to that of the Illumina-PacBio hybrid assembly contiguity (Table 4). As sequencing accuracy and throughput reliability improve and with the development of long read assemblers, this platform is headed towards stand-alone long-read assemblies that are suitable for accurate representations of microbial community structure and predicted function in complex environmental samples.

## Methods

### Cultivation and DNA extraction

Cultures of *Micromonospora coxensis* DSM 45161, *Micromonospora echinaurantiaca* DSM 43904, and *Micromonospora echinofusca* DSM 43913 were grown aerobically in DSMZ medium 65 Gym Streptomyces Medium (https://www.dsmz.de/?id=441) (DSMZ, Braunschweig, Germany) at 28 °C. Genomic DNA was isolated using the MasterPure Gram Positive DNA Purification Kit (Epicentre, Madison, WI) following the standard protocol provided by the manufacturer but modified by incubating on ice overnight on a shaker and the use of an additional 1 µl proteinase K.

Cultures of *Halomonas* sp. HL-4 and *Halomonas* sp. HL-93 were grown aerobically in Hot Lake Heterotroph (HLH) medium^59^ at 30 °C. Genomic DNA was isolated using phenol-chloroform extraction as previously described^60^.

Cultures of *Thioclava* sp. ES.032, *Propionibacteriaceae bacterium* ES.041, *Cohaesibacter* sp. ES.047, and *Muricauda* sp. ES.050 were grown aerobically on modified PE agar plates^61^. Biomass from 1–2 plates was scraped and genomic DNA was isolated using the Qiagen bacterial extraction protocol for the Genomic-tip 500/G kit (Qiagen, Germantown, MD), with minor modifications. Briefly, in addition to the buffer B1, proteinase K and RNase additions, an enzyme cocktail composed of 500 ml achromopeptidase (10 U/ml), 500 ml lysostaphin (0.2 U/ml), 500 ml of lysozyme (100 mg/ml) and 1 ml mutanolysin (1 U/ml) was added to the samples. Samples were placed on a shaker and incubated at 37 °C overnight to lyse the cells. Genomic DNA was extracted the next day using the genomic-tips 500/G, as per the manufacturer’s instructions.

The *Marinobacter* and *Psychrobacter* strains isolated from Antarctic Lake Vida (*Marinobacter* sp. LV10R510-8, *Marinobacter* sp. LV10MA510-1, and *Psychrobacter* sp. LV10R520-6) were grown aerobically in R2A media (Difco) with 5% NaCl (25 mL each) under non-shaking conditions at 10 °C. Cells were pelleted by centrifuging for 5 minutes at 12,000 × g. High molecular weight genomic DNA was isolated following Ausubel^62^. Briefly, cells were resuspended in TE buffer with 10% SDS and proteinase K (final concentration) then following 1 hr. incubation at 37 °C, CTAB (hexadecyltrimethylammonium bromide)/NaCl was added to extract the nucleic acids, and chloroform: isoamylalcohol was used to purify the preparation. The crude extract was digested with RNAse and then the HMW gDNA was precipitated in isopropanol, and following drying, the pellet was resuspended in TE.

All DNA extracts were checked for quality and quantified using a Qubit fluorometer (Invitrogen, Carlsbad, CA) and visually by quantitative gel. Samples were pooled at varying ratios from 1.6–16.2% to generate the mock community (Table 1).

### Library creation and sequencing

For Illumina library creation, 100 ng of genomic DNA, brought up to a total of 100 μl in TE, was sheared to 300 bp using the Covaris LE200 (Covaris, Inc. Woburn, MA, USA) and size-selected using SPRI beads (Roche Holding AG, Basel, Switzerland): 60 μl of beads were added to 100 μl of sample. The sample was then incubated at room temperature (RT) for 5 min. Beads were pelleted using a magnetic particle concentrator (MPC) (Thermo Fisher Scientific, South San Francisco, CA, USA) until liquid was clear. The supernatant was removed and transferred to a new tube. AMPure XP (30 μl) beads were then added for the second bead size selection. The mixture was pulse vortexed, quickly spun and incubated at RT for 5 min. Beads were pelleted using an MPC until liquid was clear. The supernatant was then discarded without disturbing the beads and 200 μl of freshly prepared 75% ethanol (EtOH) was added, followed by a 30 s incubation to wash the beads. EtOH was discarded before the EtOH wash step was repeated twice. Afterwards, the sample was placed on a thermocycler (Eppendorf, Hamburg, Germany) with the lid open and incubated at 37 °C until the beads were dry and residual EtOH had evaporated. The beads were re-suspended in 53 μl of EB buffer (Qiagen, Redwood City, CA, USA), vortexed, quickly spun and incubated at RT for 1 min. Beads were pelleted using an MPC until liquid was clear and then 50 μl of supernatant was transferred to a new tube. The fragments were treated with the Kapa Library Preparation Kit ORIGIN (Kapa Biosystems, Wilmington, MA, USA) for the following steps: For end-repair 26 μl MilliQ water, 9 μl 10X End Repair Buffer, and 5 μl End Repair Enzyme were combined in a 1.5 ml tube. The cocktail was vortexed and quickly spun, stored on ice, and then 40 μl was added to the 50 μl DNA sample. The mixture was vortexed and quickly spun, before incubation at 30 °C for 30 min in a thermocycler (Eppendorf, Hamburg, Germany). After incubation, 126 μl of AMPure XP beads (Beckman Coulter, Brea, CA, USA) were added to 90 μl of End Repair sample, pulse vortexed, quickly spun, and incubated at RT for 5 min. Beads were pelleted using an MPC until liquid was clear. The supernatant was then discarded without disturbing the beads. The beads were washed twice with 200 μl of freshly prepared 75% EtOH with an incubation time of 30 s. After washing, the sample was incubated at 37 °C in a thermocycler with the lid open until residual EtOH had evaporated. For DNA resuspension, 17.5 μl of EB buffer was added. The sample was vortexed, quickly spun, and incubated at RT for 1 min, before beads were pelleted on an MPC. 15 μl of supernatant was then transferred to a new tube.

For A-tailing, 9 μl of MilliQ water, 3 μl of 10X A-Tailing Buffer and 3 μl of A-Tailing Enzyme were combined in this order in a 1.5 ml tube. The cocktail was vortexed and quickly spun, then 15 μl of the A-Tailing cocktail was added to the 15 μl sample. The mixture was vortexed and quickly spun before incubating the samples in a thermocycler at 30 °C for 30 min, followed by 5 min at 70 °C.

Adapter ligation was performed immediately thereafter: 9 μl of 5X Ligation Buffer and 5 μl of ligase were combined in a 1.5 ml tube. The mixture was pulse vortexed and quickly spun before adding 14 μl of adapter ligation cocktail to the 30 μl sample; 1 μl of 18 μM adapter was then added to the ligation mixture for a final concentration of 400 nM. The mixture was incubated in a thermocycler at 20 °C for 15 min. After adapter ligation, 5 μl of EB Buffer was added to 45 μl of adapter-ligated sample. The sample was size-selected and washed twice with 45 μl of AMPure XP beads as described previously. After the first clean-up step, the sample was resuspended with 52 μl of EB Buffer and 45 μl of supernatant was transferred to a clean tube. After the second clean-up step, the sample was eluted with 25 μl of EB Buffer and 23 μl of supernatant was transferred to a clean tube. The sample was quality-controlled and quantified using an Agilent Bioanalyzer 2100 High Sensitivity Kit.

The prepared Illumina library was further quantified using KAPA Biosystem’s next generation sequencing library qPCR kit (Roche Holding AG, Basel, Switzerland) and run on a Roche Light Cycler 480 real-time PCR instrument according to the manufacturer’s guidelines (Roche Holding AG, Basel, Switzerland). The quantified library was then prepared for sequencing on the Illumina HiSeq sequencing platform (Illumina, Inc., San Diego, CA, USA). First, the TruSeq paired-end cluster kit, v3, and Illumina’s cBot instrument were used to generate a clustered flowcell for sequencing (Illumina, Inc., San Diego, CA, USA). Sequencing of the flowcell was performed on the Illumina HiSeq 2500 sequencer using a TruSeq SBS sequencing kit 200 cycles, v4, following a 2 × 150 indexed run recipe (Illumina, Inc., San Diego, CA, USA) (Table 2). This resulted in 426,735,646 raw reads.

For PacBio library creation, an unamplified library was generated using Pacific Biosciences standard template preparation protocol for creating >10 kb libraries. gDNA (10 μg) was sheared using Covaris g-Tubes to generate >10 kb fragments (Covaris, Inc., Woburn, MA, USA). The sheared DNA fragments were then prepared according to the Pacific Biosciences SMRTbell template preparation kit guidelines (Pacific Biosciences, Menlo Park, CA, USA). Briefly, DNA fragments were treated with DNA damage repair mix, end-repaired, and 5′ phosphorylated. PacBio hairpin adapters were then ligated to the fragments to create SMRTbell templates for sequencing. The SMRTbell templates were purified using exonuclease treatments and size-selected using the Sage Science BluePippin instrument with a 10 kb lower cutoff depending on DNA quality.

PacBio sequencing primers were annealed and v. P6 sequencing polymerase was bound to the SMRTbell templates. The prepared SMRTbell template libraries were then sequenced on a Pacific Biosciences RSII sequencer using v. C4 chemistry and 1 × 240 min sequencing movie run times (Pacific Biosciences, Menlo Park, CA, USA).

For the size-selected ONT library, 10 µg of gDNA was used and quality controlled using FA12 DNA QC. The DNA was sheared using Covaris g-Tubes to generate >10 kb fragments (Covaris, Inc., Woburn, Ma, USA). The sheared DNA fragments were then size selected using the Sage Science BluePippin instrument with a 10 kb lower cutoff. After clean-up, DNA was repaired and end-prepared using the NEBNext FFPE DNA Repair kit (New England BioLabs, Ipswich, MA, USA) with the following changes to the manufacturer’s protocol: The reaction volume was doubled to 120 µl, incubation was performed at 20 °C for 20 minutes and at 65 °C for 20 minutes. AMPure XP beads (120 µl) were added to the repaired DNA and incubated at RT for 30 minutes on a Hula mixer, followed by two washes with 70% EtOH. Beads were then resuspended with 61 µl of nuclease-free (NF) water and incubated at RT for 30 minutes on a Hula mixer; 61 µl of the eluate was then transferred into a clean 1.5 ml Eppendorf tube. The resulting DNA was quantified using the Qubit HS DNA kit.

Adapter ligation and clean-up was performed using the Ligation Sequencing Kit SQK-LSK109 (Oxford Nanopore Technologies, Oxford, United Kingdom) with a slightly changed protocol: Ligation buffer, NEBNext Quick T4 DNA ligase, and adapter mix were added to the repaired DNA and incubated at RT for 10 minutes and then overnight at 4 °C. The ligated sample was purified using 100 µl of AMPure XP beads during a 30 minute incubation at RT on the Hula mixer, two bead washing steps using the kit-provided wash buffer and resuspension of the beads in 40 µl of elution buffer at RT for 30 minutes on the Hula mixer; 40 µl of the eluate was then transferred into a clean 1.5 ml tube.

The library was then sequenced on a MinION using R9.4.1 flow cell sequencing chemistry (Table 2). This resulted in 187,507 Pass-1D reads that were processed using the MinKNOW software version 1.13.1.

For the non-size-selected ONT library, 5 μg of gDNA was used to create the ONT library. The DNA was sheared using Covaris g-tubes to generate >10 kb fragments (Covaris Inc., Woburn, MA USA). The sheared DNA was repaired using the NEBNext FFPE Repair Mix (New England BioLabs, Ipswich, MA USA) according to the manufacturer’s instructions. AMPure XP beads (62 μl) were added to the FFPE-repair reaction and incubated at RT for 30 minutes on a Hula mixer, followed by two washes with 70% EtOH. Beads were then resuspended with 93 μl of NF water and incubated for 30 minutes at room temperature on a Hula mixer; 90 μl of the eluate was then transferred to a clean 1.5 mL Eppendorf tube. The resulting DNA was quantified using the Qubit HS DNA kit.

The fragmented and repaired DNA underwent end repair and A-tailing using the NEBNExt End Repair/dA-Tailing Module (New England BioLabs) with the following changes to the manufacturer’s protocol: The reaction volume was doubled to 120 μl, incubation was performed at 20 °C for 20 minutes and at 65 °C for 20 minutes. AMPure XP beads (120 μl) were added to the end-prep reaction and incubated for 30 minutes at room temperature on a Hula mixer, followed by two washes with 70% EtOH. Beads were then resuspended in 31 ul of NF water and incubated for 30 minutes at room temperature on a Hula mixer; 61 μl of the eluate was then transferred to a clean 1.5 mL Eppendorf tube. The resulting DNA was quantified using the Qubit HS DNA kit.

Adapter ligation and clean-up was performed using the SQK-LSK108 kit (Oxford Nanopore Technologies, Oxford, United Kingdom) with the following changes to the manufacturer’s protocol: The ligation reaction was incubated at room temperature for 10 minutes and then overnight at 4 °C. The ligated samples were purified using 40 μl of AMPure XP beads, incubated for 30 minutes at room temperature on a Hula mixer followed by two washes using the kit-provided wash buffer. The beads were resuspended in 15 μl of the kit-provided elution buffer and then incubated for 30 minutes at room temperature on a Hula mixer; 15 μl of the eluate was then transferred to a clean 1.5 mL tube and quantified using the Qubit HS DNA kit.

The library was then sequenced on a MinION using the R9.4 flow cell sequencing chemistry and resulted in 144,976 reads.

### Sequence QC

BBDuk (filterk = 27 trimk = 27; https://sourceforge.net/projects/bbmap/) was used to remove Illumina adapters, known Illumina artifacts, and phiX, and to quality-trim both ends to Q12 from the Illumina library. Reads were discarded if they contained more than one ‘N’, or had quality scores (before trimming) averaging less than 8 over the read, or had a length under 40 bp after trimming. The remaining reads were mapped to a masked version of human HG19, dog, cat, and mouse with BBMap (https://sourceforge.net/projects/bbmap/), discarding all hits over 93% identity. This process yielded 422,896,888 filtered reads (Table 2). Quality filtering of PacBio sequences were performed using SMRT Portal v2.3.0, setting minimum subread length to 50, minimum polymerase read quality to 75, minimum polymerase read length to 50, and control spike-in was removed using pbalign with parameters minAccuracy = 0.75 minLength = 50. Filtering yielded 389,806 subreads. ONT basecalling was performed using Albacore basecaller v2.3.1 selecting only the pass-1D reads.

### Read Mapping and repeat region identification

Illumina, PacBio, and ONT reads were mapped to reference genomes using bwa v0.7.15 (http://bio-bwa.sourceforge.net/) with default parameters for Illumina. Parameters -x pacbio and -x ont2d were specified for PacBio and ONT reads, respectively. The number of reads that mapped to *Micromonospora coxensis* was negligible. The distribution of reads that mapped to each organism, as well as numbers of reads that did not map to any organism, are given in Table S1. Reference sequences were downloaded from IMG on June 27, 2017. IMG IDs for references are listed in Table 1. Repeats in genomes were found using repeat-match tool from MUMmer package v3.23^63^, specifying parameter -n25.

### Assembly and assembly quality assessment

For the assembly, we first performed error correction on Illumina reads using bfc version r181 with parameters -1 -s 10 g -k 21 -t 10^64^. Unpaired reads were removed from the library subsequently. Error-corrected reads were then assembled using SPAdes v3.12.0^65^ with parameters -m 120 –only-assembler -k 33,55,77,99,127 –meta. For the hybrid assemblies, ONT and PacBio reads were supplied to the assembler via–nanopore and–pacbio parameters. Long reads were not error corrected as recommended in the SPAdes manual. Assembly statistics were generated using metaquast from Quast 4.6.3^66^ package using default parameters.

### Data post-processing

Depth of coverage plots in Figs. 3 and S7 were produced using bedtools genomecov^67^. Illumina insert size distribution in Fig. S6 was obtained using picard CollectInsertSizeMetrics^68^. We used jgi_summarize_bam_contig_depths (bitbucket.org/berkeleylab/metabat) with parameter–percentIdentity 70 to produce GC coverage plots in Fig. S8. Percent identity distributions in Figs. S3, S4, error rates in Fig. S9, and distributions in Fig. S10 were generated using jgi_summarize_bam_contig_depths (bitbucket.org/berkeleylab/metabat). Figures S11 and S12 were produced from Metaquast output.

The bash scripts used for QC, mapping, assembly and post-processing are available at https://bitbucket.org/volkansevim/bmock12/src/master/.

## Data Records

Shotgun sequences generated on the Illumina, ONT, and PacBio platforms are publicly available through NCBI and details are listed in Supplementary Table 6: SRA Accessions SRX5161985^69^ (ONT no size selection), SRX4901586^70^ (ONT 10 kb size selection), SRX4901584^71^ & SRX4901585^72^ (PacBio 10 kb size selection; two libraries were combined for analysis), SRX4901583^73^ (Illumina). Assemblies have been deposited at NCBI Assembly under the accessions GCA_003957615.1^74^ (PacBio + Illumina hybrid), GCA_003957625.1^75^ (ONT + Illumina hybrid), and GCA_003957645.1^76^ (Illumina only).

## Technical Validation

To assess the quality of genomic DNA received, we used the PicoGreen assay and the Qubit 2.0 fluorometer (Invitrogen, Carlsbad, CA, USA). Each sample was quantified in quadruplicate.

## Supplementary information

Supplementary Figures

Supplementary Tables
