# Supplementary material for: Shotgun metagenome data of a defined mock community using Oxford Nanopore, PacBio and Illumina technologies
Source: Sci Data. 2019 Nov 26;6:285. doi: 10.1038/s41597-019-0287-z (PMC6879543; doi:10.1038/s41597-019-0287-z)
Supplement: Supplementary file 1 — Supplementary Figures [file 41597_2019_287_MOESM1_ESM.pdf]

List of Figures

1 Length distribution of mapped reads from non-size selected ONT library. . . . 2
2 % Mapped bases distribution for the ONT libraries. . . . 2
3 % Identity distribution of mapped ONT and PacBio reads. . . . 2
4 % Identity distribution of mapped long-reads. . . . 3
5 Length distribution of long reads. . . . 3
6 Length distribution of mapped reads. . . . 4
7 Distribution of sequencing depth per organism. . . . 5
8 GC bias. . . . 6
9 Error rates in long read mappings. . . . 7
10 Difference between the read length vs aligned length for long reads. . . . 7
11 NGA50, LGA50 and misassembled contigs length distributions. . . . 8
12 Dot plots for all assemblies. . . . 9

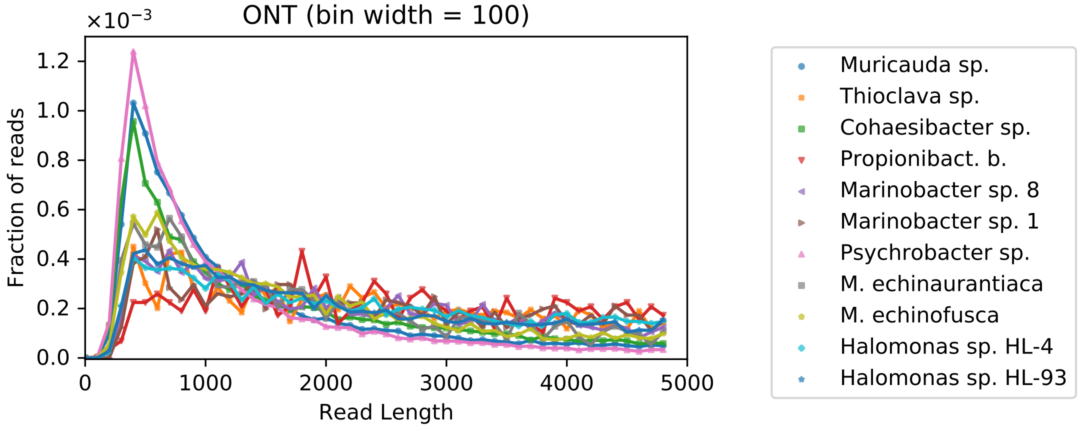

Fig. S1: Length distribution of mapped reads from non-size selected ONT library. Connecting lines are guides to the eye.

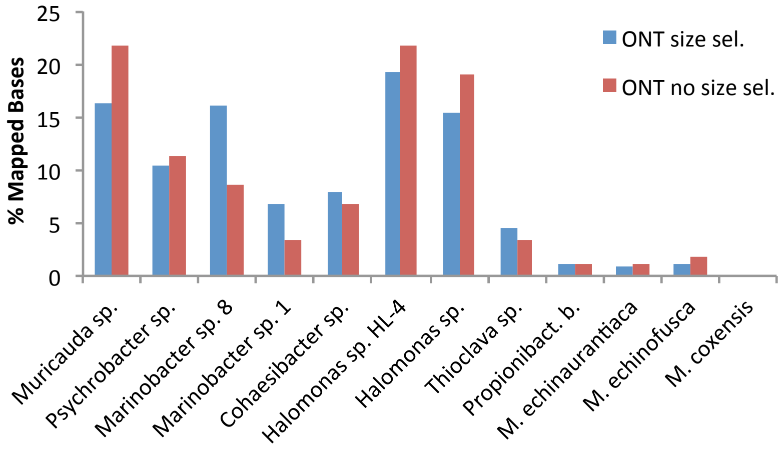

Fig. S2: % Mapped bases distribution for the ONT libraries with and without size selection.

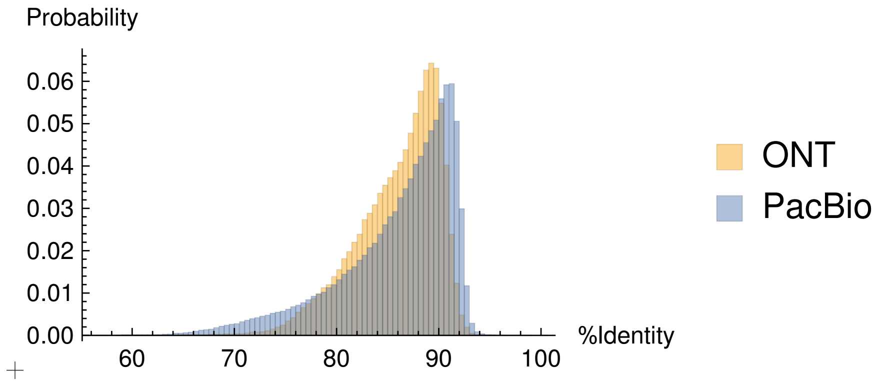

Fig. S3: % Identity distribution of mapped ONT and PacBio reads. Both distributions have the same mean, 85.9%. Standard deviations are 0.057 and 0.042 for PacBio and ONT, respectively.

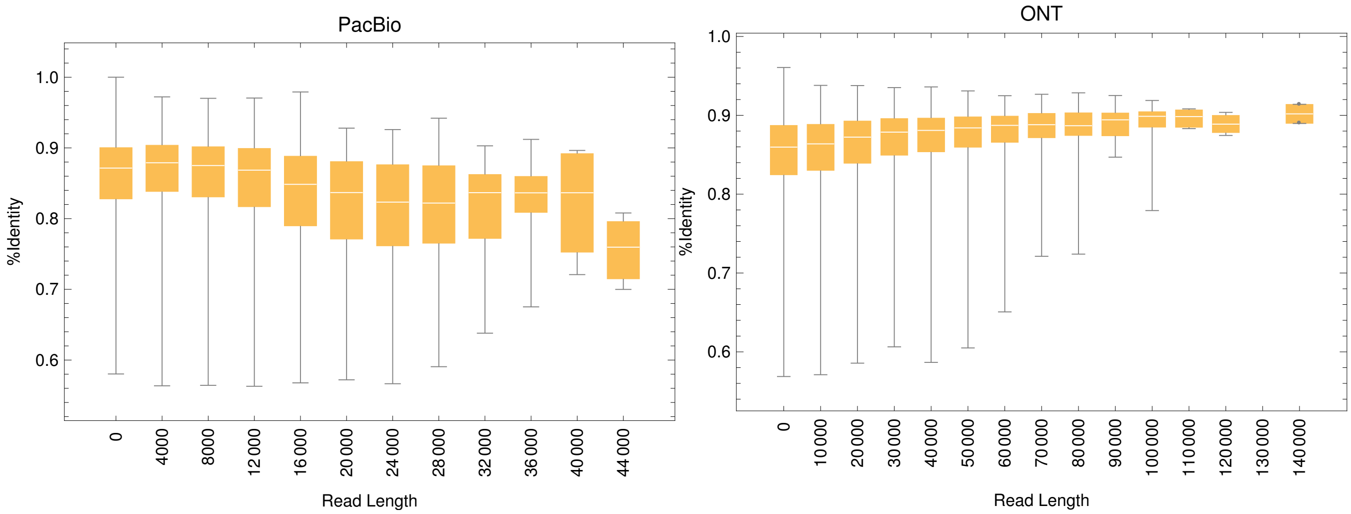

Fig. S4: % Identity distribution of mapped long-reads, binned by length.

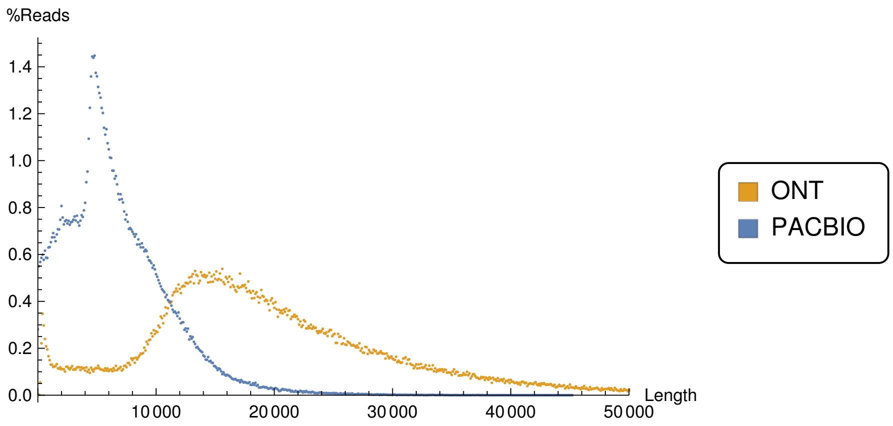

Fig. S5: Length distribution of long reads.

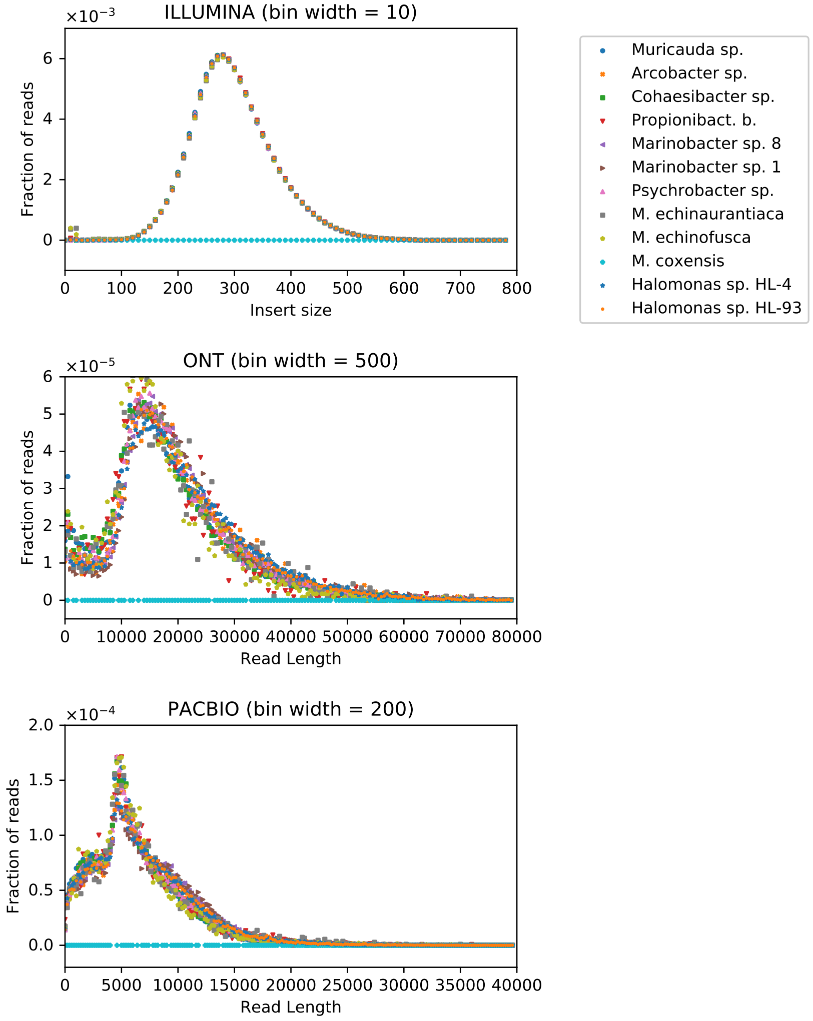

Fig. S6: Length distribution of mapped reads, normalized for each organism.

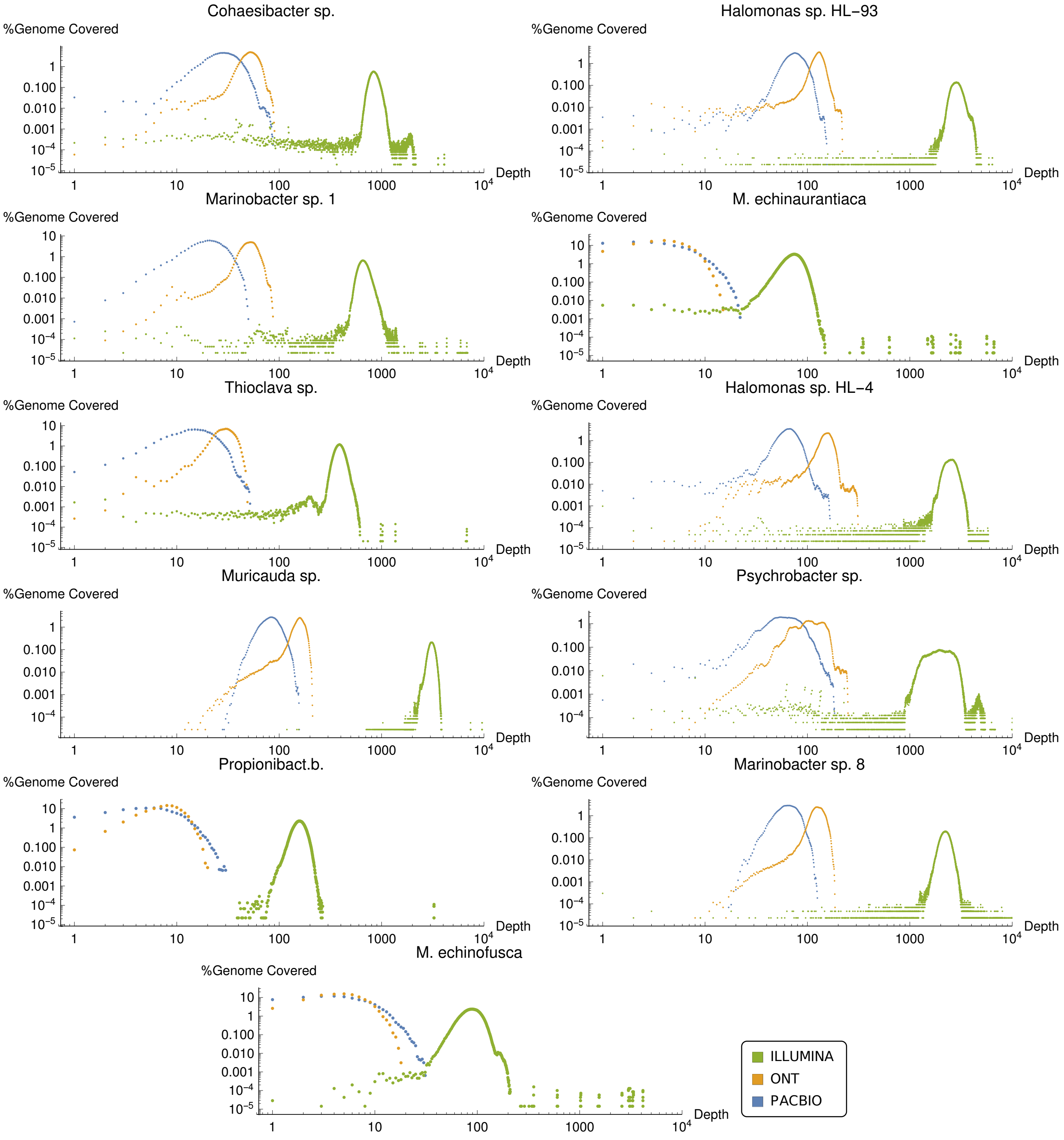

Fig. S7: Distribution of sequencing depth per organism for all three technologies.

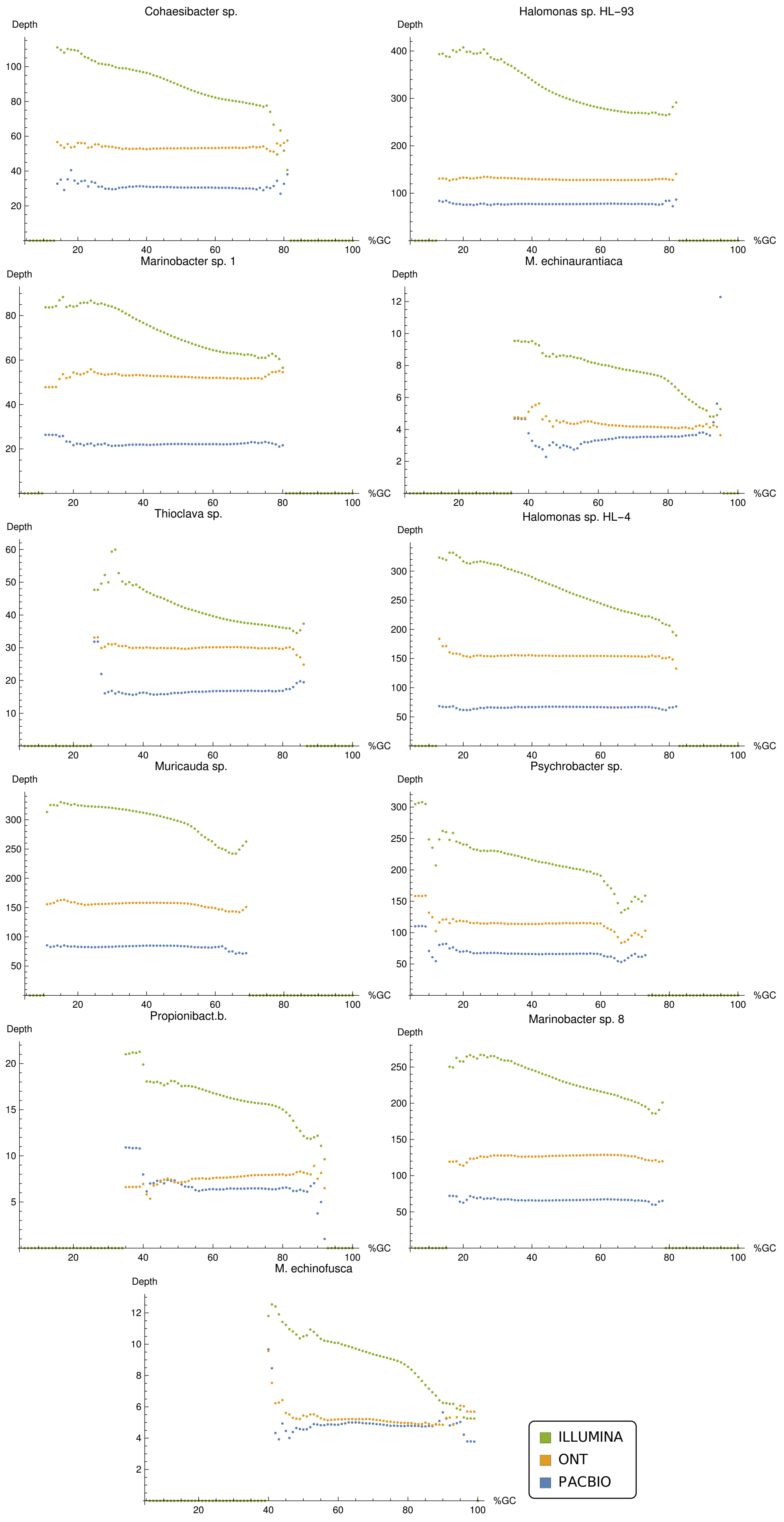

Fig. S8: GC bias. Illumina depth was scaled by 0.1.

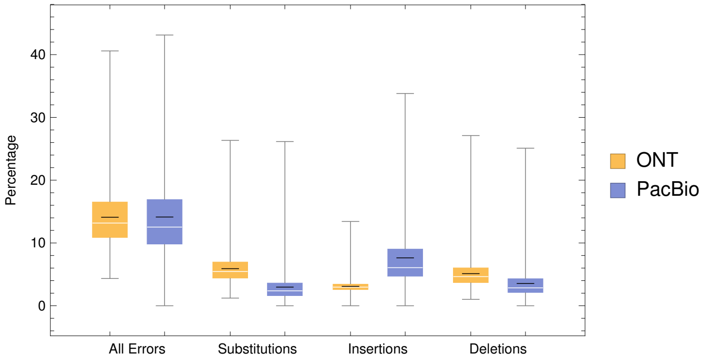

Fig. S9: Error rates in long read mappings. Yellow boxes mark 25%, median, and the 75%, black bars mark minima, mean and maxima. Means for PacBio reads are 14.1, 2.97, 7.64, 3.54, means for ONT reads are 14.1, 5.9, 3.09, 5.1, for all errors, substitutions, insertions, and deletions, respectively. All Errors percantage corresponds to $100(S+D+I)/(S+D+I+E)$, where S, D, I, and E stand for substitutions, deletions, insertions, and exact matches, respectively.

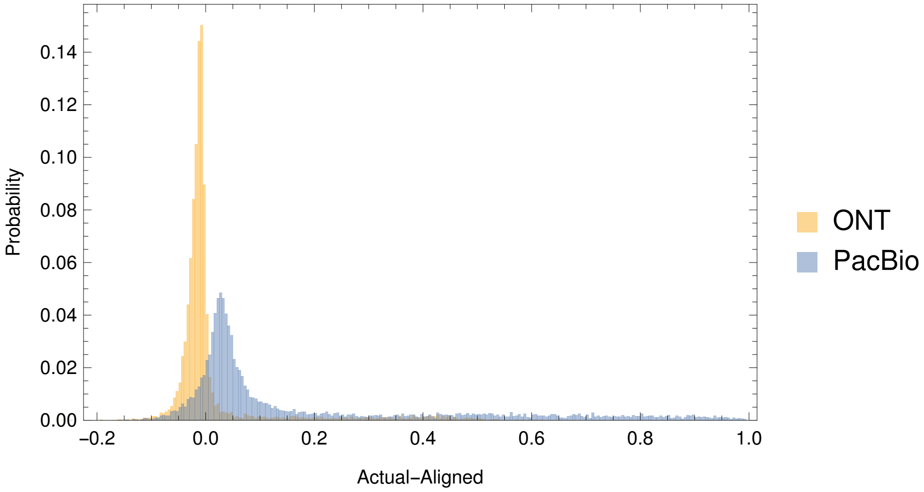

Fig. S10: Difference between the read length and the aligned length (normalized by read length) for long reads.

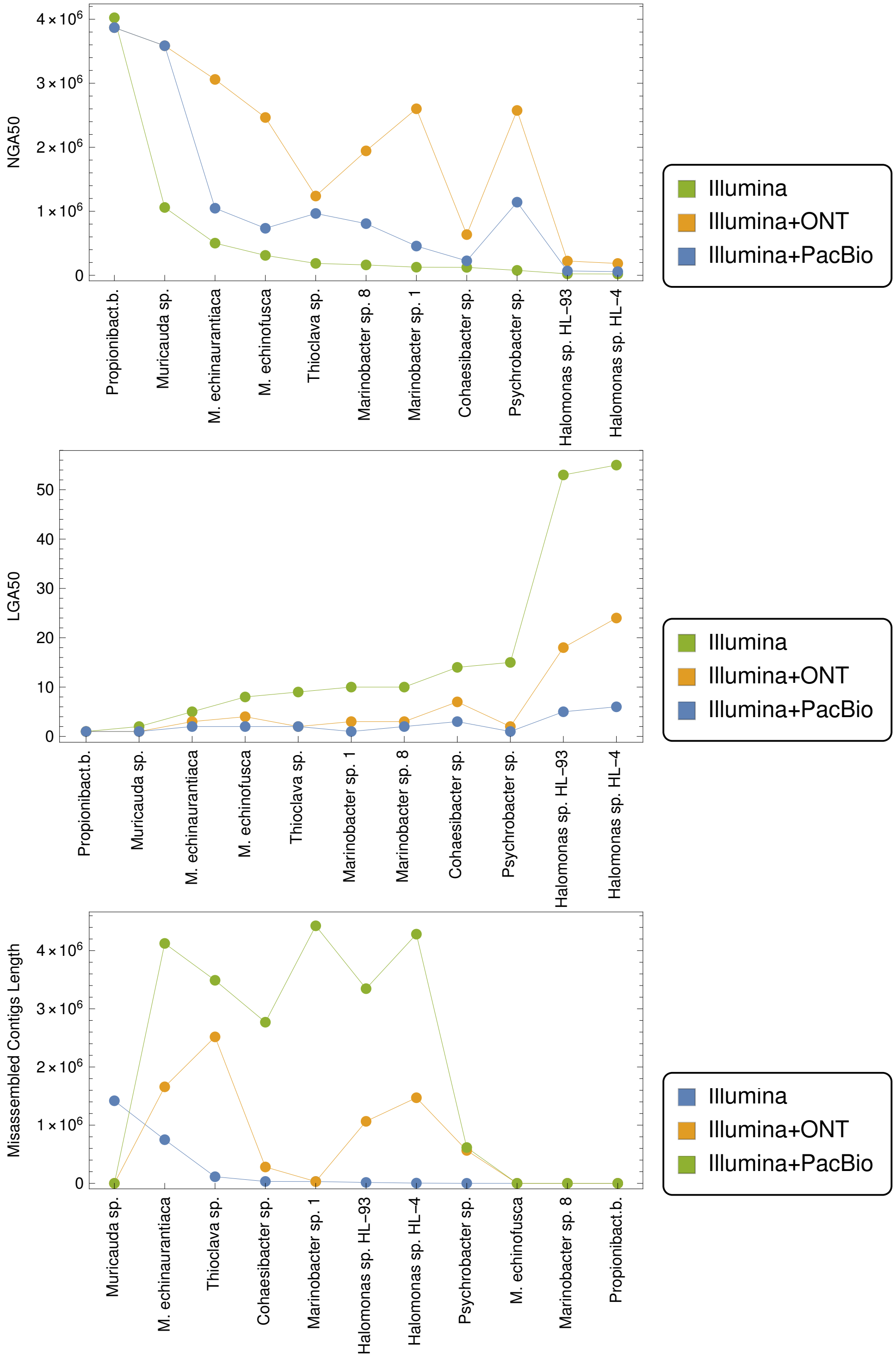

Fig. S11: NGA50, LGA50 and misassembled contigs length distributions for all assemblies. Connecting lines are guides to the eye.

Muricauda sp. ES.050

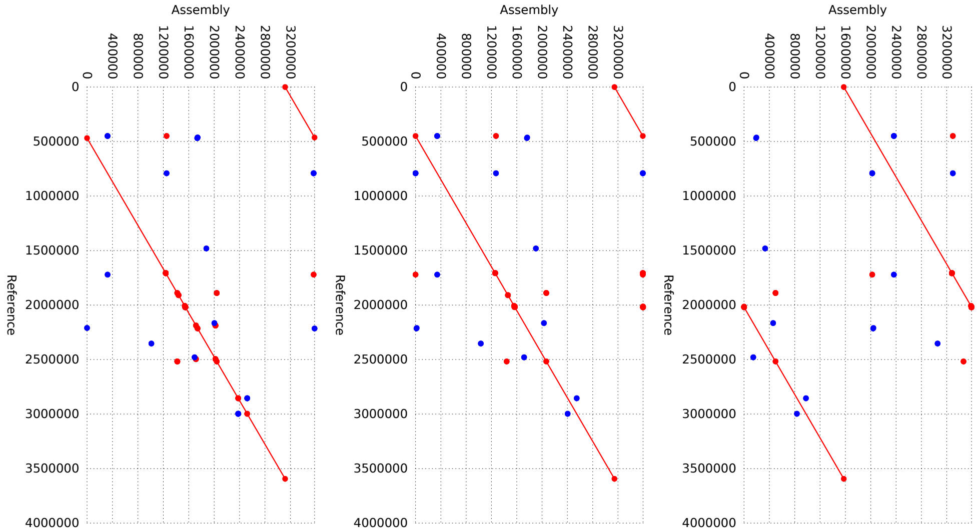

Thioclava sp. ES.032

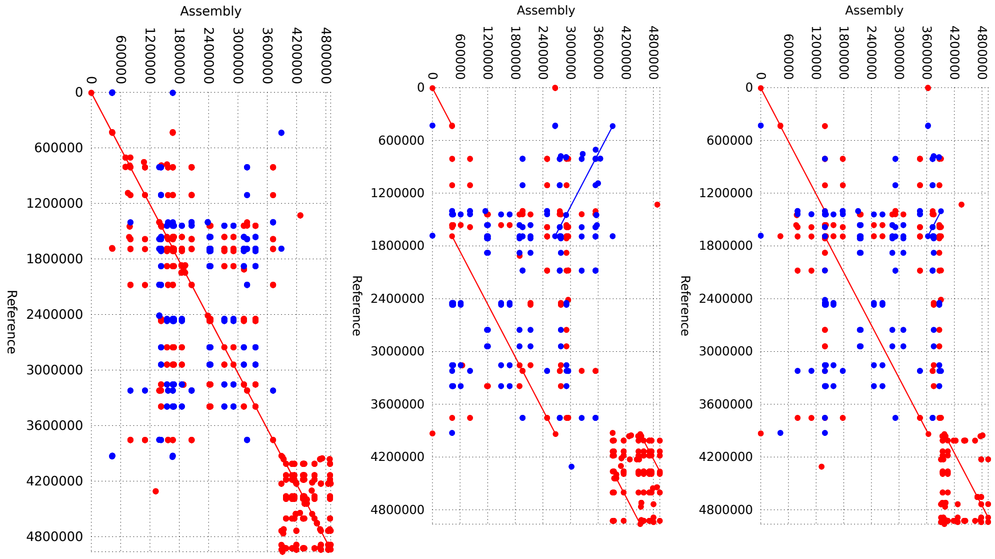

Cohaesibacter sp. ES.047

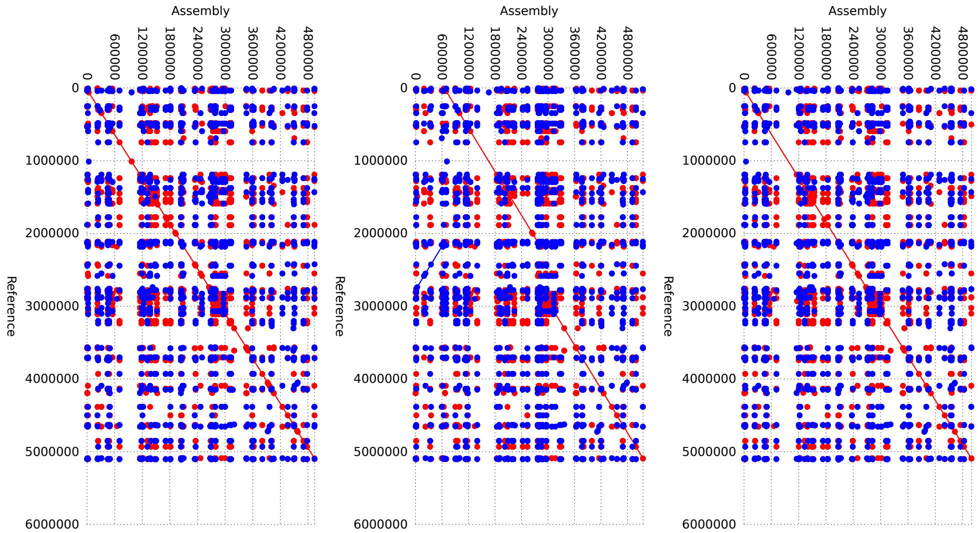

Fig. S12: Dot plots for all assemblies. Illumina, ONT+Illumina, Pacbio+Illumina, on the left, center, and right, repectively. Red/blue dots and lines indicate forward/reverse matches. (Figure continued on next three pages.)

Propionibacteriaceae bacterium ES.041

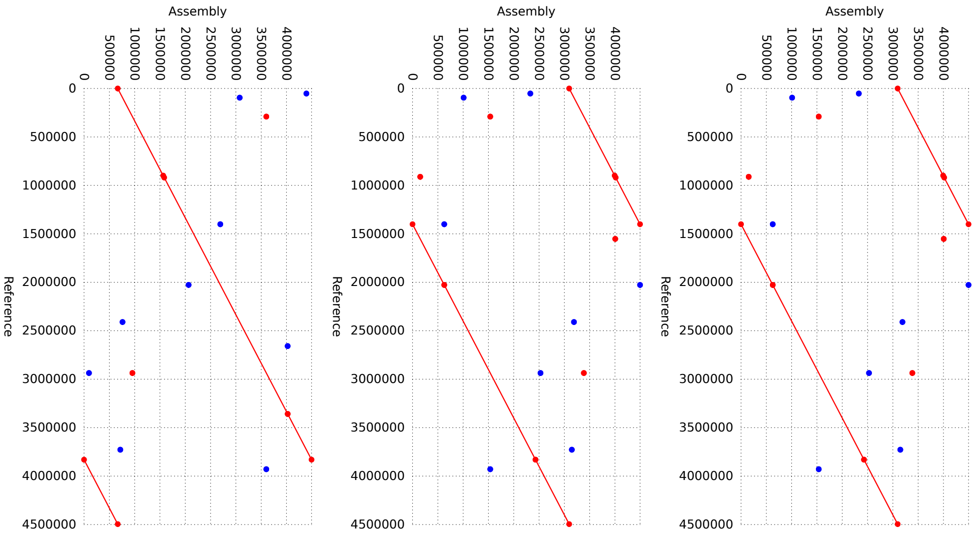

Marinobacter sp. LV10R510-8

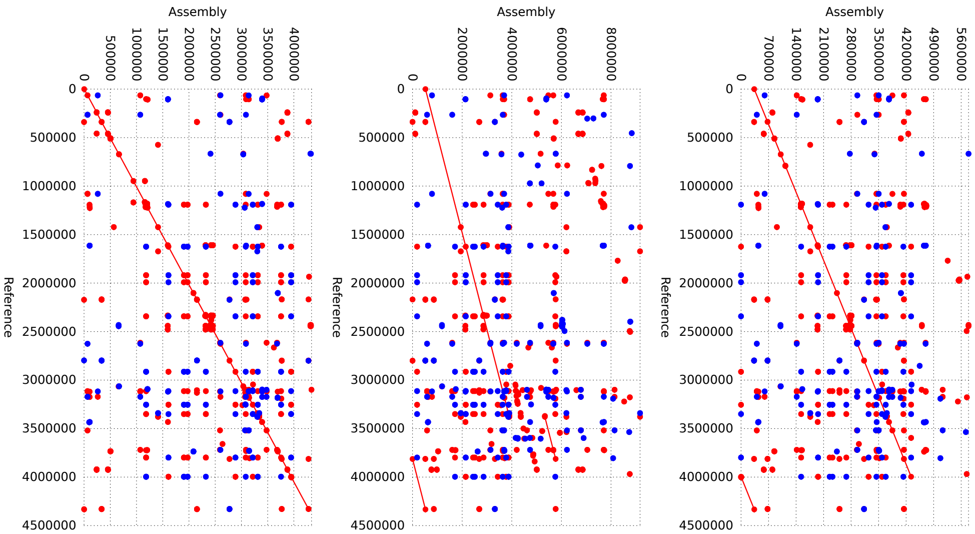

Marinobacter sp. LV10MA510-1

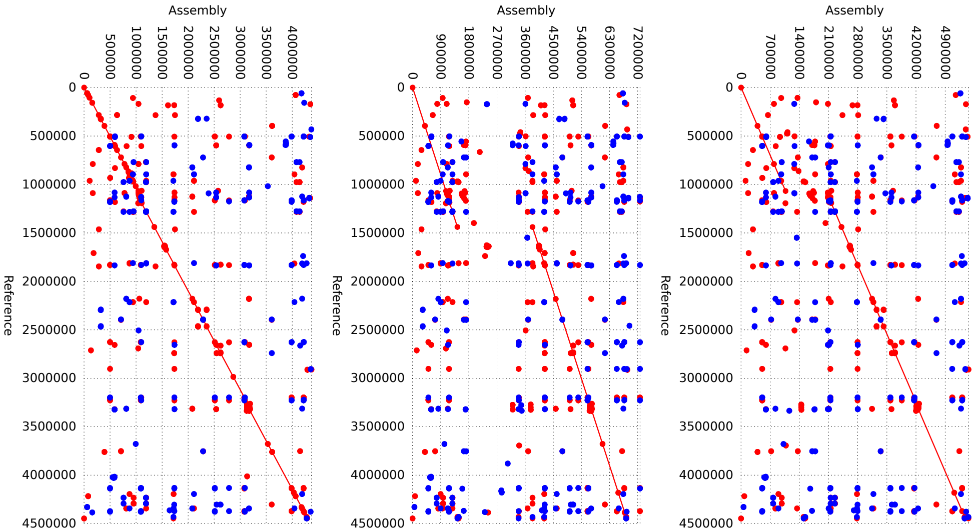

Figure S12 (continued)

Psychrobacter sp. LV10R520-6

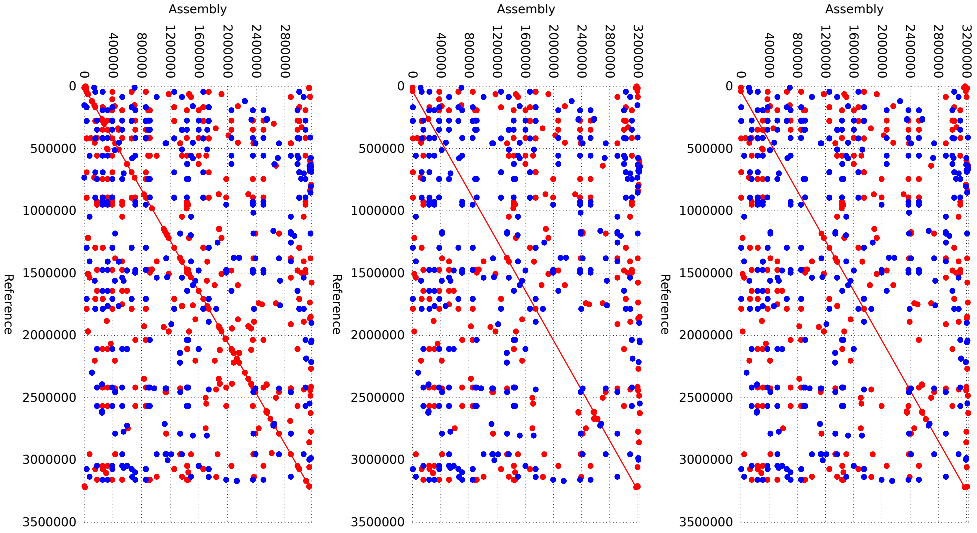

Micromonospora echinaurantiaca DSM 43904

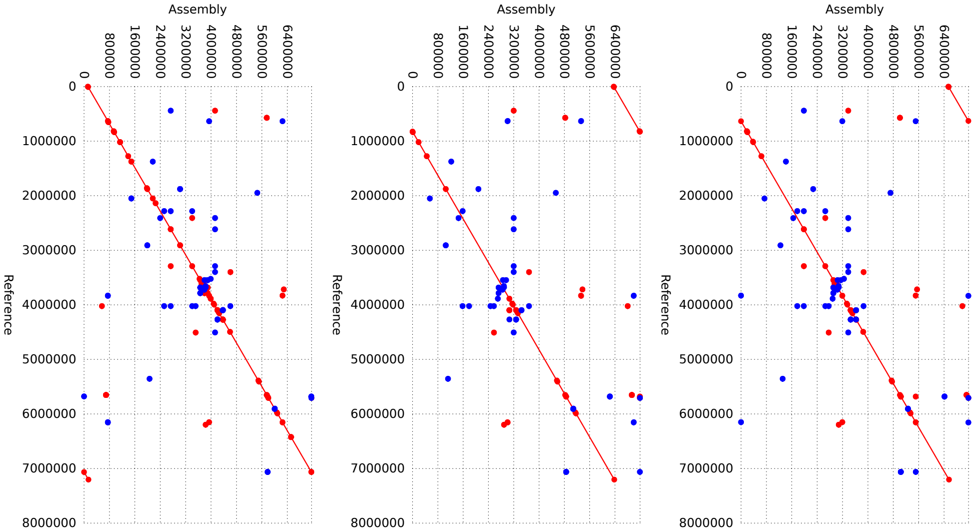

Micromonospora echinofusca DSM 43913

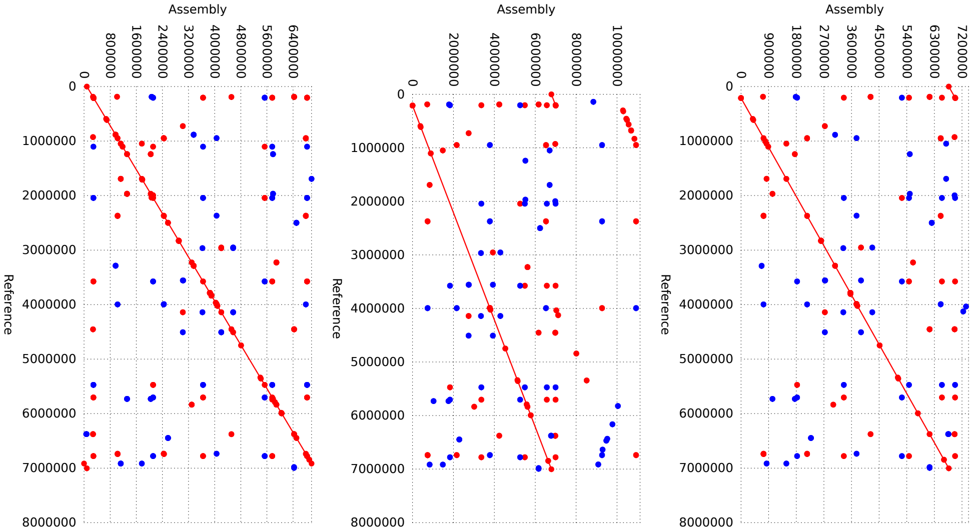

Figure S12 (continued)

Halomonas sp. HL-4

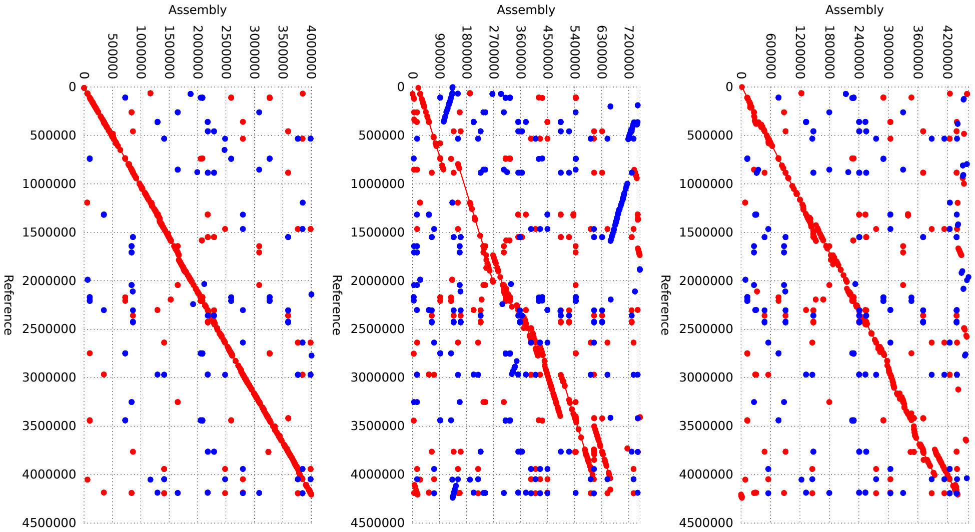

Halomonas sp. HL-93

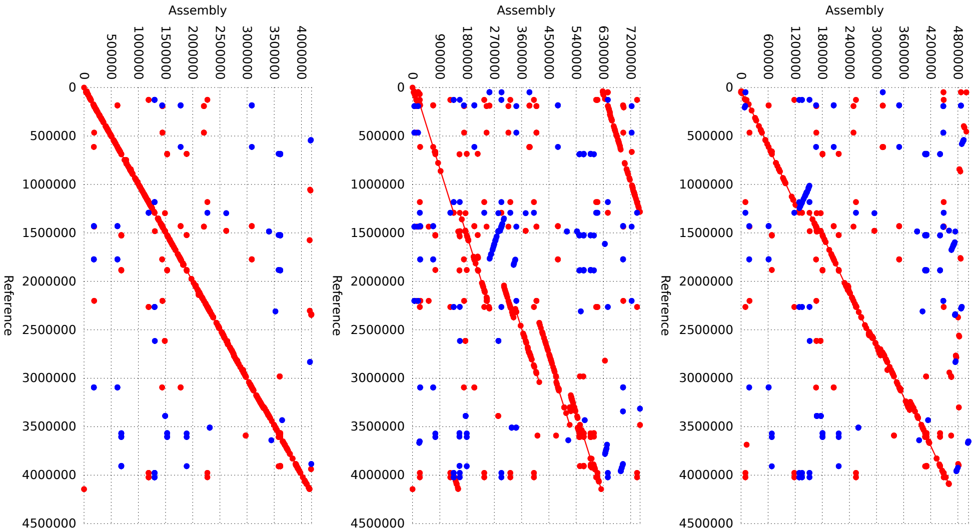

Figure S12 (continued)
