# Supplementary material for: Shotgun metagenome data of a defined mock community using Oxford Nanopore, PacBio and Illumina technologies
Source: Sci Data. 2019 Nov 26;6:285. doi: 10.1038/s41597-019-0287-z (PMC6879543; doi:10.1038/s41597-019-0287-z)
Supplement: Supplementary file 2 — Supplementary Tables [file 41597_2019_287_MOESM2_ESM.docx]

**Table of Contents**

1. Table S1.  Reference statistics obtained from the IMG database. 2
2. Table S2. Numbers of mapped reads to each genome by each technology. 2
3. Table S3. Numbers of mapped bases to each genome by each technology. 3
4. Table S4: Mash distances between genomes. 4
5. Table S5: Supplementary assembly statistics 5

Table S1.  Reference statistics obtained from the IMG database. All genomes are available as improved high-quality drafts. Genome size was used to normalize molarity calculations.

| **IMG Taxon ID** | **Organism** | **No.**  **scaf-**  **folds** | **Genome**  **size [bp]** | **% GC** | **% Repeat** | **Molarity**  **×10^-15^** |
| --- | --- | --- | --- | --- | --- | --- |
| 2615840527 | *Muricauda* sp. ES.050 | 1 | 3,593,735 | 40.3 | 1.5 | 5.95 |
| 2615840533 | *Thioclava* sp. ES.032 | 2 | 4,962,820 | 62.8 | 3.44 | 1.61 |
| 2615840601 | *Cohaesibacter* sp. ES.047 | 1 | 5,110,232 | 55.5 | 7.27 | 3.06 |
| 2615840646 | *Propionibacteriaceae* bacterium ES.041 | 1 | 4,495,552 | 69.5 | 2.42 | 0.74 |
| 2615840697 | *Marinobacter* sp. LV10R510-8 | 1 | 4,333,333 | 53.9 | 4.8 | 7.10 |
| 2616644829 | *Marinobacter* sp. LV10MA510-1 | 1 | 4,447,234 | 54.6 | 5.83 | 3.37 |
| 2617270709 | *Psychrobacter* sp. LV10R520-6 | 1 | 3,218,993 | 42.9 | 7.23 | 7.18 |
| 2623620557 | *Micromonospora echinaurantiaca* DSM 43904 | 1 | 7,203,133 | 73.2 | 2.91 | 0.47 |
| 2623620567 | *Micromonospora echinofusca* DSM 43913 | 1 | 7,002,527 | 73.3 | 3.85 | 0.43 |
| 2623620609 | *Micromonospora coxensis* DSM 45161 | 1 | 6,769,693 | 73.6 | 3.48 | 0.77 |
| 2623620617 | *Halomonas* sp. HL-4 | 1 | 4,238,582 | 55.8 | 4.35 | 7.26 |
| 2623620618 | *Halomonas* sp. HL-93 | 1 | 4,144,442 | 55.9 | 2.78 | 7.42 |

Table S2. Numbers of mapped reads to each genome by each technology.

| **Organism** | **ILLUMINA** | **PACBIO** | **ONT** |
| --- | --- | --- | --- |
| Muricauda sp. | 74,263,482 | 67,142 | 33,138 |
| Psychrobacter sp. | 45,769,662 | 44,980 | 20,373 |
| Marinobacter sp. 8 | 64,862,842 | 55,432 | 29,916 |
| Marinobacter sp. 1 | 20,090,318 | 18,868 | 11,999 |
| Cohaesibacter sp. | 29,119,402 | 33,154 | 15,913 |
| Halomonas sp. HL-4 | 71,778,728 | 57,501 | 33,548 |
| Halomonas sp. HL-93 | 80,284,678 | 63,094 | 28,133 |
| Thioclava sp. | 12,987,850 | 16,675 | 7,955 |
| Propionibact. b. | 4,794,346 | 6,394 | 2,293 |
| M. echinaurantiaca | 3,614,040 | 5,538 | 1,824 |
| M. echinofusca | 4,279,722 | 7,787 | 2,346 |
| M. coxensis | 18,442 | 18 | 10 |
| **TOTAL MAPPED** | **411,863,512** | **376,583** | **187,448** |
| **TOTAL READS** | 422,896,888 | 389,806 | 187,507 |
| **UNMAPPED** | 11,033,376 | 13,223 | 59 |

Table S3. Numbers of mapped bases to each genome by each technology.

| **Organism** | **ILLUMINA** | **PACBIO** | **ONT** |
| --- | --- | --- | --- |
| Muricauda sp. | 11,118,249,065 | 305,116,246 | 567,117,705 |
| Psychrobacter sp. | 6,847,954,294 | 214,164,181 | 368,591,918 |
| Marinobacter sp. 8 | 9,703,090,034 | 289,361,566 | 554,787,364 |
| Marinobacter sp. 1 | 3,003,004,257 | 98,869,129 | 232,582,333 |
| Cohaesibacter sp. | 4,358,693,593 | 156,592,035 | 271,908,425 |
| Halomonas sp. HL-4 | 10,746,508,657 | 284,283,625 | 654,685,792 |
| Halomonas sp. HL-93 | 12,016,274,981 | 322,368,280 | 531,758,938 |
| Thioclava sp. | 1,943,367,674 | 83,733,782 | 149,536,017 |
| Propionibact. b. | 717,097,216 | 31,035,149 | 37,920,358 |
| M. echinaurantiaca | 537,056,259 | 28,996,402 | 32,751,261 |
| M. echinofusca | 636,915,104 | 38,941,335 | 38,305,537 |
| M. coxensis | 645,893 | 161,457 | 185,849 |
| **TOTAL MAPPED** | **61,628,857,027** | **1,853,623,187** | **3,440,131,497** |
| **TOTAL BASES IN FASTQ** | **63,384.840,109** | **2,583,337,248** | **3,737,495,058** |

Table S4: Mash distances between genomes. d<0.1 marked in red, 0.1<d<0.2 in yellow, 0.2<d<0.3 in green. Mash distance is correlated with average nucleotide identity (ANI). See reference in the main text.

|  | Muricauda sp. | Thioclava sp. | Cohaesibacter sp. | Propionibact. b. | Marinobacter sp. 8 | Marinobacter sp. 1 | Psychrobacter sp. | M. echinaurantiaca | M. echinofusca | M. coxensis | Halomonas sp. HL-4 | Halomonas sp. HL-93 |
| --- | --- | --- | --- | --- | --- | --- | --- | --- | --- | --- | --- | --- |
| Muricauda sp. |  |  |  |  |  |  |  |  |  |  |  |  |
| Thioclava sp. | 1.00 |  |  |  |  |  |  |  |  |  |  |  |
| Cohaesibacter sp. | 1.00 | 0.41 |  |  |  |  |  |  |  |  |  |  |
| Propionibact. b. | 0.41 | 1.00 | 0.41 |  |  |  |  |  |  |  |  |  |
| Marinobacter sp. 8 | 1.00 | 1.00 | 1.00 | 1.00 |  |  |  |  |  |  |  |  |
| Marinobacter sp. 1 | 1.00 | 1.00 | 0.41 | 0.41 | 0.15 |  |  |  |  |  |  |  |
| Psychrobacter sp.6 | 1.00 | 1.00 | 1.00 | 1.00 | 0.35 | 0.35 |  |  |  |  |  |  |
| M. echinaurantiaca | 1.00 | 0.37 | 0.41 | 0.28 | 1.00 | 1.00 | 1.00 |  |  |  |  |  |
| M. echinofusca | 1.00 | 0.35 | 1.00 | 0.28 | 1.00 | 1.00 | 1.00 | 0.11 |  |  |  |  |
| M. coxensis | 1.00 | 0.37 | 0.41 | 0.28 | 1.00 | 1.00 | 1.00 | 0.12 | 0.12 |  |  |  |
| Halomonas sp. HL-4 | 1.00 | 0.37 | 0.41 | 1.00 | 0.41 | 0.33 | 0.41 | 1.00 | 1.00 | 0.41 |  |  |
| Halomonas sp. HL-93 | 1.00 | 0.35 | 0.41 | 0.41 | 0.41 | 0.33 | 0.41 | 1.00 | 0.37 | 0.41 | 0.01 |  |

Table S5: Supplementary assembly statistics generated by Metaquast. LGA50 is the minimum number of aligned blocks that cover half the reference genome. Aligned blocks are obtained by breaking contigs at misassembly events and removing all unaligned bases. Refer to Quast manual for detailed explanations of the metrics. (<http://quast.bioinf.spbau.ru/manual.html>)

|  |  | **Total**  **Aligned**  **Length (bp)** |  |  | **Largest Contig** |  |  | **LGA50** |  |
| --- | --- | --- | --- | --- | --- | --- | --- | --- | --- |
| **Assemblies** | **Illumina Only** | **Illumina +ONT** | **Illumina +PacBio** | **Illumina Only** | **Illumina +ONT** | **Illumina +PacBio** | **Illumina Only** | **Illumina +ONT** | **Illumina +PacBio** |
| Muricauda sp. | 3,579,680 | 3,588,800 | 3,588,688 | 1,419,363 | 3,593,028 | 3,587,960 | 2 | 1 | 1 |
| Thioclava sp. | 4,897,995 | 4,936,485 | 4,929,955 | 424,883 | 3,490,222 | 2,517,750 | 9 | 2 | 2 |
| Cohaesibacter sp. | 4,943,183 | 5,037,148 | 4,978,611 | 265,743 | 1,790,557 | 676,632 | 14 | 3 | 7 |
| Propionibact. b. | 4,495,270 | 4,495,746 | 4,495,746 | 4,023,073 | 3,869,214 | 3,869,214 | 1 | 1 | 1 |
| Marinobacter sp. 8 | 4,279,897 | 4,466,641 | 4,355,418 | 325,327 | 2,992,736 | 1,142,095 | 10 | 2 | 3 |
| Marinobacter sp. 1 | 4,305,045 | 4,507,397 | 4,435,065 | 370,982 | 2,992,736 | 1,142,095 | 10 | 1 | 3 |
| Psychrobacter sp. | 3,167,398 | 3,222,825 | 3,218,178 | 164,730 | 2,575,453 | 1,208,012 | 15 | 1 | 2 |
| M. echinaurantiaca | 7,159,006 | 7,186,693 | 7,165,977 | 1,183,569 | 4,123,941 | 1,788,945 | 5 | 2 | 3 |
| M. echinofusca | 6,965,319 | 7,045,622 | 6,978,679 | 723,771 | 4,123,941 | 1,627,731 | 8 | 2 | 4 |
| Halomonas sp.HL-4 | 3,995,790 | 5,559,163 | 4,515,191 | 88,380 | 852,999 | 294,361 | 55 | 6 | 24 |
| Halomonas sp.HL-93 | 4,176,376 | 5,461,159 | 4,740,445 | 88,380 | 852,999 | 294,361 | 53 | 5 | 18 |
| not_aligned |  |  |  | 9,958 | 9,958 | 9,958 |  |  |  |
|  |  | **Misassem-**  **bled**  **contigs**  **Length (bp)** |  |  | **#Indels**  **per 100kbp** |  |  | **#Mismatches**  **per**  **100 kbp** |  |
| **Assemblies** | **Illumina Only** | **Illumina +ONT** | **Illumina +PacBio** | **Illumina Only** | **Illumina +ONT** | **Illumina +PacBio** | **Illumina Only** | **Illumina +ONT** | **Illumina +PacBio** |
| Muricauda sp. | 1,419,363 | - | - | 0.36 | 0.36 | 0.36 | 3.49 | 3.57 | 3.57 |
| Thioclava sp. | 113,694 | 3,490,222 | 2,517,750 | 0.41 | 0.87 | 0.97 | 0.59 | 3.16 | 3.47 |
| Cohaesibacter sp. | 32,773 | 2,769,727 | 281,196 | 0.16 | 0.22 | 0.18 | 1.21 | 6.2 | 3.66 |
| Propionibact. b. | - | - | - | 1.36 | 1.27 | 1.27 | 0.02 | 0.02 | 0.02 |
| Marinobacter sp. 8 | - | - | - | 0.77 | 0.92 | 0.88 | 3.39 | 10.13 | 8.28 |
| Marinobacter sp. 1 | 30,937 | 4,427,028 | 30,937 | 0.72 | 2.62 | 1.55 | 13.47 | 78.97 | 61.02 |
| Psychrobacter sp. | 658 | 615,431 | 565,796 | 0.1 | 2.57 | 2.32 | 2.36 | 12.69 | 11.45 |
| M. echinaurantiaca | 750,126 | 4,123,941 | 1,658,856 | 0.61 | 0.65 | 0.59 | 3.13 | 3.88 | 3.73 |
| M. echinofusca | - | - | - | 0.47 | 0.4 | 0.54 | 2.24 | 2.27 | 2.01 |
| Halomonas sp. HL-4 | 4,150 | 4,283,361 | 1,543,806 | 3.39 | 10.96 | 10.66 | 273.72 | 565.3 | 579.35 |
| Halomonas sp. HL-93 | 15,420 | 3,373,358 | 1,065,743 | 1.01 | 5.32 | 3.45 | 30.8 | 213.05 | 130.25 |
